# Effects of NAD^+^ in *Caenorhabditis elegans* Models of Neuronal Damage

**DOI:** 10.3390/biom10070993

**Published:** 2020-07-02

**Authors:** Yuri Lee, Hyeseon Jeong, Kyung Hwan Park, Kyung Won Kim

**Affiliations:** 1 Department of Life Science, Hallym University, Chuncheon 24252, Korea; m20019@hallym.ac.kr (Y.L.); m20021@hallym.ac.kr (H.J.); m20017@hallym.ac.kr (K.H.P.); 2 Convergence Program of Material Science for Medicine and Pharmaceutics, Hallym University, Chuncheon 24252, Korea; 3 Multidisciplinary Genome Institute, Hallym University, Chuncheon 24252, Korea

**Keywords:** NAD^+^, Nmnat, NMAT-2, PARP, *C. elegans*, neuroprotection, axon regeneration

## Abstract

Nicotinamide adenine dinucleotide (NAD^+^) is an essential cofactor that mediates numerous biological processes in all living cells. Multiple NAD^+^ biosynthetic enzymes and NAD^+^-consuming enzymes are involved in neuroprotection and axon regeneration. The nematode *Caenorhabditis elegans* has served as a model to study the neuronal role of NAD^+^ because many molecular components regulating NAD^+^ are highly conserved. This review focuses on recent findings using *C. elegans* models of neuronal damage pertaining to the neuronal functions of NAD^+^ and its precursors, including a neuroprotective role against excitotoxicity and axon degeneration as well as an inhibitory role in axon regeneration. The regulation of NAD^+^ levels could be a promising therapeutic strategy to counter many neurodegenerative diseases, as well as neurotoxin-induced and traumatic neuronal damage.

## 1. NAD*^+^* Biosynthesis Pathway in *C. elegans*

**Figure 1:**
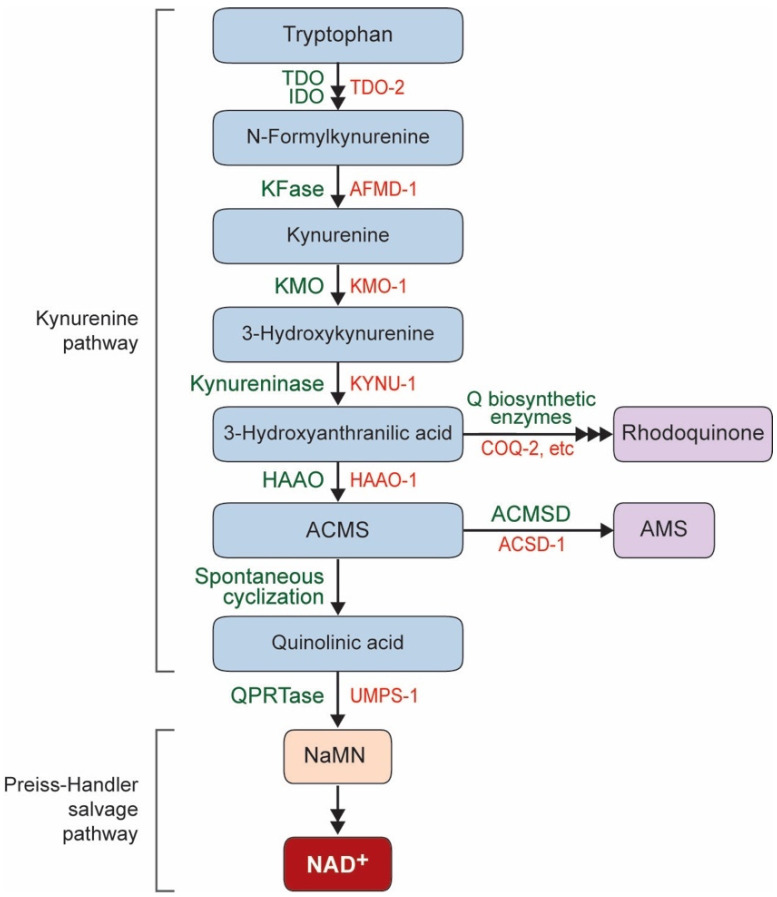
The de novo Nicotinamide adenine dinucleotide (NAD^+^) biosynthesis pathway starts with the degradation of the essential amino acid tryptophan via the kynurenine pathway and produces quinolinic acid, which merges with the Preiss–Handler salvage pathway and produces NAD^+^. TDO, Tryptophan-2,3-dioxygenase; IDO, Indoleamine-2,3-dioxygenase; KFase, Kynurenine formamidase; KMO, Kynurenine-3-monooxygenase; HAAO, 3-hydroxyanthranilate 3,4-dioxygenase; ACMS, α-amino-β-carboxymuconate-ε-semialdehyde; AMS, α-aminomuconate semialdehyde; ACMSD, ACMS decarboxylase; QPRTase, QA phosphoribosyltransferase. Green text: general enzyme names or events; red text: *C. elegans* genes encoding corresponding enzymes.

Nicotinamide adenine dinucleotide (NAD^+^) is found in all living cells and plays an essential role in many fundamental biological processes, such as metabolism, cell signaling, gene expression, and DNA repair [1]. NAD^+^ is synthesized through two metabolic pathways, either a de novo pathway or salvage pathways. In a de novo pathway, NAD^+^ can be synthesized from the degradation of the essential amino acid L-tryptophan via the kynurenine pathway (Figure 1) [2,3]. The derivatives of the kynurenine pathway have been linked to both the progression and protection of neurological disorders and neurodegenerative diseases [4]. The kynurenine pathway produces quinolinic acid (QA), which is converted to nicotinic acid mononucleotide (NaMN) by QA phosphoribosyltransferase (QPRTase) and merges with the Preiss–Handler salvage pathway [5]. QA is a known neurotoxin [6,7]; thus, a proper clearing mechanism should be fulfilled by QPRTase. Although the de novo pathway is conserved in *C. elegans*, no QPRTase homolog has been found [8]. A recent study showed that the enzyme uridine monophosphate phosphoribosyltransferase (UMPS), encoded by the *umps-1* gene in *C. elegans*, possesses QPRTase activity [9]. A lack of *umps-1* in *C. elegans* results in decreased levels of global NAD^+^ and increased steady-state levels of QA, indicating that UMPS-1 is required for NAD^+^ de novo synthesis in place of the missing QPRTase in *C. elegans* [9]. Thus, the de novo NAD^+^ biosynthesis pathway is functionally conserved in *C. elegans.* However, the neuronal roles of the kynurenine pathway in *C. elegans* are yet to be determined.

**Figure 2:**
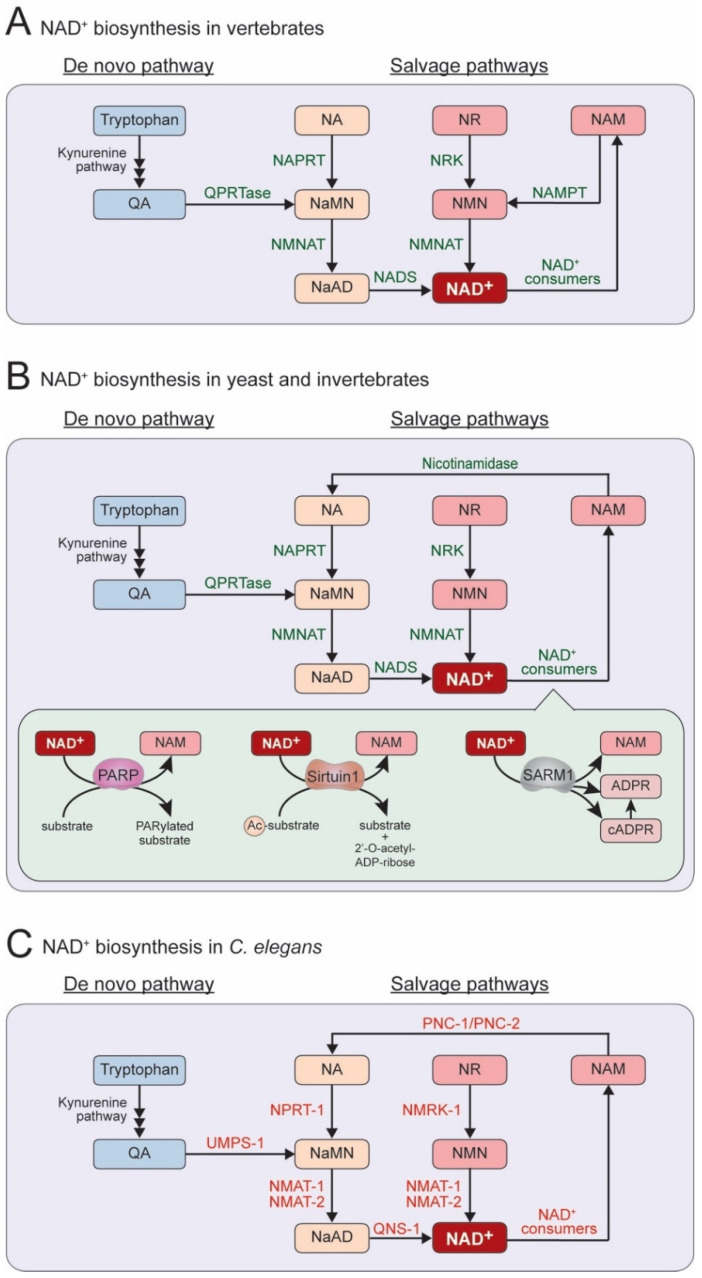
NAD^+^ biosynthesis pathway in (**A**) vertebrates, (**B**) yeast and invertebrates, and (**C**) *C. elegans*. PARylated, Poly Adenosine diphosphate (ADP)-Ribosylated; ADPR, ADP-ribose; cADPR, cyclic ADPR.

Alternatively, NAD^+^ can be produced from NAD^+^ precursors, which include nicotinic acid (NA), nicotinamide riboside (NR), and nicotinamide (NAM) (Figure 2A–C) [10,11]; the salvage synthesis from NA is termed the Preiss–Handler pathway [12,13]. These three compounds are termed vitamin B_3_ or niacin and can be taken up from the diet. These compounds are also produced within cells as intermediates of NAD^+^ biosynthesis. NAD^+^ is consumed and converted to NAM by various enzymes, such as poly(ADP-ribose) polymerases (PARPs), sirtuins, and Sarm1 [8,14]. At least two types of NAM salvage pathways are known: vertebrates use a one-step pathway, whereas yeast and invertebrates use a two-step pathway. Vertebrates convert NAM into nicotinamide mononucleotide (NMN) by Nicotinamide phosphoribosyltransferase (NAMPT), which is then converted to NAD^+^ by Nicotinamide mononucleotide adenylyltransferase (NMNAT) (Figure 2A). In primitive eukaryotes, including yeast, *C. elegans*, and *Drosophila*, NAMPT activity has not been found [8]. In these species, NAM is converted to NA using a nicotinamidase, which then enters the Preiss–Handler pathway (Figure 2B,C).

Despite the presence of the de novo pathway, the salvage pathways are essential in animals. Interestingly, in *C. elegans*, mutants lacking components of the de novo pathway show normal viability, while some mutants lacking components of the salvage pathways show lethality. So far, two genes are found to be essential for viability, *nmat-2* (Nicotinamide Mononucleotide AdenylylTransferase-2) and *qns-1* (Glutamine-dependent NAD^+^ Synthase-1) [15]. *nmat-2* encodes a *C. elegans* NMNAT homolog, and *qns-1* encodes a NAD^+^ synthase (NADS) homolog. The homozygous *nmat-2* or *qns-1* offspring of heterozygous mutants are viable but sterile, whereas homozygous mutants produce no offspring [15], suggesting that maternally or paternally derived NAD^+^ is likely critical for embryogenesis. In contrast, the other enzymes in either de novo or salvage pathways (the protein encoded by *tdo-2*, *afmd-1*, *kmo-1*, *haao-1*, *umps-1*, *pnc-1*, *pnc-2*, *nprt-1*, *nmat-1*, or *nmrk-1*) are found to be non-essential for animal survival, although some mutants show a low fecundity. Thus, in *C. elegans*, NMAT-2 and QNS-1 are likely rate-limiting or nonredundant among the components in the NAD^+^ biosynthesis pathways.

## 2. Neuroprotective Effect of NAD^+^ in *C. elegans*

**Figure 3:**
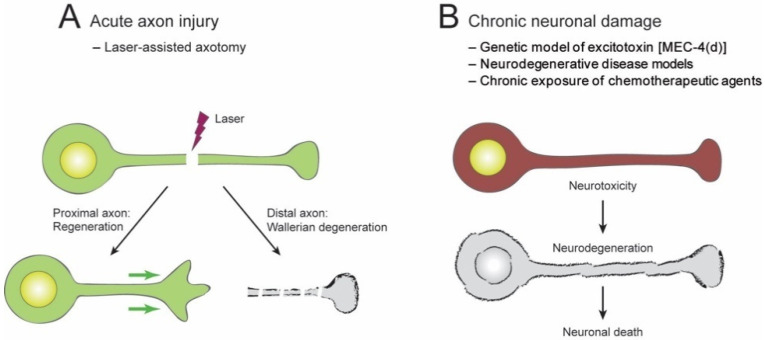
*C. elegans* models of neuronal damage. (**A**) Acute axon injury by laser-assisted axotomy. (**B**) Chronic neuronal damage models.

The NAD^+^ biosynthesis pathway appears to play a role in protecting neurons from damage and stresses. Depending on the duration of the harmful stimulus, the types of damage can be classified into acute (short-term) and chronic (long-term) injuries (Figure 3A,B) [16]. Acute axon injuries physically sever axons into two parts, which can be manifested by transecting neurons or laser-assisted microsurgical cutting of the individual axons. Chronic injuries result from persistent neuronal stresses such as the expression of harmful proteins or hazardous environmental conditions. In response to these acute or chronic injuries, neurons may undergo axon degeneration, cell death, or axon regeneration. These neuronal responses can vary depending on the types of damage [16].

### 2.1. Acute Axon Injuries

In an acute axon injury, the axon is severed (axotomy) into two fragments in relation to the cell body: a proximal fragment and a distal fragment. The proximal axon fragment may undergo axonal regeneration, whereas the distal axon fragment often undergoes a rapid axonal degeneration (Figure 3A). Axon degeneration is an early feature of most neuronal injuries and is a primary cause of functional impairment. The degeneration of axons distal to a lesion site is highly stereotyped and is known as Wallerian degeneration [17,18]. In this process, the distal part of an axon first experiences a latent phase. It then undergoes a catastrophic fragmentation into numerous pieces, after which the pieces are completely removed via a genetically encoded “self-destruction” program [19].

Wallerian degeneration is delayed dramatically in (1) the Wallerian degeneration slow (*Wld^s^*) mutant mice, (2) the *Sarm1* null mutant mice, and (3) *Nmnat* overexpressed mice [20,21,22,23], suggesting that the Wallerian degeneration is an active molecular mechanism. First, *Wld^s^* is a spontaneous mutant gene that encodes a fusion protein, WLD^s^, which likely has a sustained NMNAT activity in the cytosol [24]. Second, SARM1 exhibits a NADase activity [25], and SARM1 activation thus reduces NAD^+^ levels and vice versa [26,27]. Third, NMNAT is an NAD^+^-synthesizing enzyme, and loss-of-function studies suggest that NMNAT in healthy neurons plays a protective role in inhibiting axon degeneration [28,29]. These observations support the idea that sustaining high levels of NAD^+^ inhibits or delays Wallerian degeneration.

The Wallerian degeneration paradigm has also been studied in *C. elegans.* Following a laser axotomy, *C. elegans* sensory and motor neurons exhibit an axon degeneration with a morphological similarity to the Wallerian degeneration, such as axonal swelling, thinning, breaks, and clearance. However, this axon degeneration occurs independently of the WLD^s^, NMNAT, and SARM1 pathways because the overexpression of *Wld^s^* or the endogenous *Nmnat* gene, or the lack of *Sarm1* (*C. elegans* homolog, *tir-1*) showed no protective role against axon degeneration [30]. These observations suggest that high levels of NAD^+^ may not inhibit axon degeneration in all neuron types or acute injuries. Further experiments are needed to clarify this point.

### 2.2. Chronic Injuries

Chronic neuronal stressors can cause axon degeneration and excitotoxicity (Figure 3B). Chronic stressors include several neurodegenerative diseases and exposure to chronic neurotoxins such as heavy metals and chemotherapeutic drugs [31]. Exposure to low levels of chronic excitotoxin may result in a slow pathological cascade, axonal degeneration, and pathology. In many neuropathies, the axon’s most terminal connections are lost and are termed “dying back degeneration” [32,33]. Severe excitotoxicity can result in the initiation of cell death pathways.

*C. elegans* is a valuable model for studying the underlying mechanisms of chronic axon injuries. The processes of synaptic release, trafficking, and production of neurotransmitters are conserved in *C. elegans*, and the neuronal morphology, changes in gene expression and neurotransmitters, and behaviors can be examined. We summarize the function of the *C. elegans* NAD^+^ pathway in chronic neuronal injuries by stressors, including a genetic model of excitotoxin, several neurodegenerative diseases, and exposure to chemotherapeutic agents.

#### 2.2.1. Genetic Model of Neuronal Excitotoxicity

**Figure 4:**
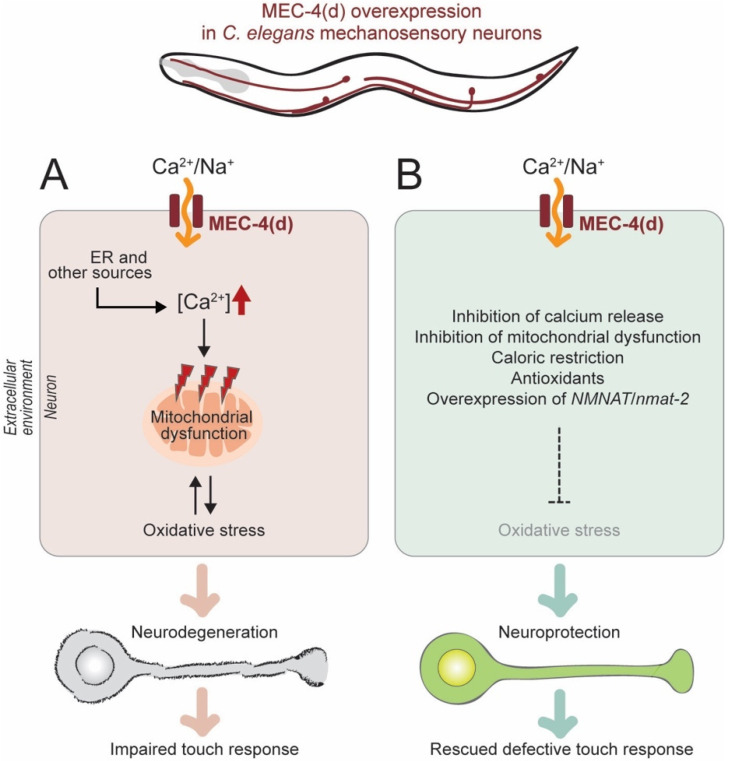
*C. elegans* models of chronic neuronal damage. (**A**) In the *mec-4(d*) excitotoxic model of *C. elegans* mechanosensory neurons, intracellular calcium increase and mitochondrial dysfunction produce oxidative stress, which triggers neurodegeneration and eventually an impaired touch response. (**B**) Relieving oxidative stress with various strategies protects neurons against degeneration and rescues touch response defects.

Neuronal excitotoxicity is often induced by an excessive release of the neurotransmitter glutamate and triggers neurite degeneration and neuronal death [34,35,36]. Hyperactivation of the degenerin channel MEC-4 (MEChanosensory abnormality-4) (MEC-4(d)) can trigger axon degeneration and the cell death of *C. elegans* neurons (Figure 4A) [37]. Stereotypically, axons become swollen and later truncated; cell bodies become vacuolated and then disappear. In the *mec-4(d)* excitotoxic model of *C. elegans* mechanosensory (touch-sensing) neurons, touch response is impaired [38]. These defects are rescued by the inhibition of calcium increase and mitochondrial dysfunction [38]. In addition, overexpression of NMNAT/NMAT-2 protects the cell body and axons against *mec-4(d)*-triggered degeneration, and enables a neuronal function such as touch response (Figure 4B) [38]. Here, reactive oxygen species (ROS) are identified as a key intermediate of neuronal degeneration triggered by MEC-4(d) stimuli. Interestingly, caloric restriction and systemic antioxidant treatment (ROS scavengers such as trolox and ascorbic acid) help decrease oxidative damage and protect both cell bodies and axons from *mec-4(d)*-triggered degeneration. However, it remains to be determined how an increase of NMNAT or NAD^+^ levels is responsible for relieving oxidative damage and playing a neuroprotective role. Interestingly, the neuroprotective effects of NMNAT seem to be variable according to the type of nerve injuries. In *C. elegans*, NMNAT is required to inhibit or delay a genetic insult-induced chronic axon degeneration, but not an injury-induced acute axon degeneration [30,38]. Additionally, the relationships between NAD^+^ levels and the regulation of calcium signaling also remain to be further studied. Advances in live-cell and in vivo imaging technology will allow us to track the changes of NAD^+^ and NAD^+^ intermediates in different subcellular compartments. They will enable us to reveal linkages among NAD^+^ levels, oxidative stress, calcium surge, and neuron degeneration.

Another *C. elegans* study using the *mec-4(d)* neurotoxic model identifies that a lack of mitochondrial sirtuin *sir-2.3*, homologous to mammalian *Sirt4*, is protective in neuronal death [39]. Sirtuins use NAD^+^ as a co-substrate in their enzymatic reaction. Interestingly, blocking the NAD^+^ salvage pathway by the knockout of nicotinamidase/*pnc-1* protects neurons from another neurotoxic model such as hypoxic ischemia with azide at low pH [39]. The neuroprotective effects seen in *sir-2.3* or *pnc-1* loss-of-function mutants are not additive, suggesting that *sir-2.3* and *pnc-1* are in a common genetic pathway. *pnc-1* mutants display a dramatic increase in NAM levels and a mild decrease in NAD^+^ levels [40]. When considering NAM as a pan-sirtuin inhibitor, although the effects of NAM are highly complicated [41], high levels of NAM in *pnc-1* mutants may inhibit SIR-2.3. Furthermore, ROS is eliminated more efficiently in animals that lack *sir-2.3* than wild types, under caloric restriction conditions. Thus, the protective effects of *sir-2.3* mutants seem to be mediated by the reduction of oxidative damage, which is similar to that seen under *nmat-2-*overexpressed conditions, suggesting that *sir-2.3* and *nmat-2* have opposite roles in neuronal protection. Therefore, it would be interesting to explore whether or how NAD^+^ augmentation in mitochondria is critical to efficiently eliminating ROS.

#### 2.2.2. *C. elegans* Models of Neurodegenerative Diseases

Neuronal excitotoxicity has been described as a contributing factor in several pathologies, including Huntington’s disease, Alzheimer’s disease, and Parkinson’s disease. *C. elegans* serves as an efficient model system for these age-dependent neurodegenerative disease studies [42].

##### Huntington’s Disease

Huntington’s disease (HD) is an inherited, autosomal, dominant neurological disorder that leads to the progressive degeneration of neurons. In HD, protein denaturation occurs because of a toxic mutation of the huntingtin protein containing an abnormally long polyglutamine (polyQ) tract. Parker and colleagues established the HD model in *C. elegans* and studied the expression of a fragment of the huntingtin gene in the mechanosensory neurons. These worms show a mutant huntingtin-dependent cytotoxicity as well as a touch response defect [43]. The upregulation of *C. elegans sir-2.1/Sirt1* recovers the axonal morphology, which in turn allows the touch response to function [43]. In addition, treatment with a sirtuin activator, resveratrol [44], shows a similar effect. Such effects are lost in *sir-2.1* mutants, indicating that the mode of action for the protection from mutant huntingtin toxicity is through SIR-2.1. Considering that NAD^+^ is the rate-limiting co-substrate for SIRT1, increased levels of NAD^+^ presumably activate SIRT1 like resveratrol; however, this remains to be determined. Conversely, as mentioned above, the knockout of a mitochondrial sirtuin *sir-2.3/Sirt4* protects neurons from *mec-4(d)*-triggered degeneration [39]. Thus, the neuroprotective effects of sirtuins are variable.

Mammalian sirtuins are NAD^+^-dependent deacylases, and there are seven sirtuin homologs (SIRT1–SIRT7) with varied subcellular localizations [45]. SIRT1 in the brain exerts neuroprotection against ischemic injury and neurodegenerative diseases, such as HD [46]. In the mouse model of HD, SIRT1 activation by resveratrol reduces the peripheral nerve deficits. However, it leads to no significant improvement in central nervous system deficits such as motor impairment and striatal atrophy [47]. SIRT1 mediates neuroprotection in mice HD models through activation of the brain-derived neurotrophic factor expression [48]. Additionally, the brain-specific knockout of *Sirt1* exacerbates the brain pathology, and the overexpression of *Sirt1* improves neuronal survival [49]. In contrast to SIRT1, the closest homolog of SIRT1, SIRT2, is detrimental to HD, because inhibition of SIRT2 protects neurons in animal models of HD [50,51]. These results again show the variable effects of sirtuins in neuroprotection.

In fly HD models, the genetic and pharmacological reduction of the *Drosophila* SIRT1 homolog results in the clearance of mutant huntingtin and neuroprotection [52]. Based on this finding, clinical trials using Selisistat (an inhibitor of SIRT1) [53] to counter the toxicity induced by huntingtin were conducted, but no clinically relevant effects have been published [54,55,56]. Currently, no therapeutics are available to treat HD.

##### Alzheimer’s Disease

A study using another *C. elegans* model of neurodegenerative disease, the Alzheimer’s disease (AD) model [57,58,59], showed a link between NAD^+^ levels and mitophagy induction [60]. Mitophagy, the removal of damaged mitochondria through autophagy, is impaired in AD patients and the *C. elegans* AD model [60]. The typical pathology of AD is the accumulation of damaged mitochondria [61]. *C. elegans* models of AD have been established by overexpressing amyloid-β or tau proteins. One of the NAD^+^ precursors, NR, reduces amyloid-β proteotoxicity in both *C. elegans* and mouse models of AD [62]. Mitophagy stimulation via supplementation with another NAD^+^ precursor, NMN, reverses memory impairment in the *C. elegans* model of AD [60]. Mitophagy enhancement also abolishes AD-related tau hyperphosphorylation in human neurons and reverses memory impairment in transgenic tau mice [60]. These results suggest that the supplementation of NAD^+^ precursors may help remove defective mitochondria and reduce proteotoxicity in AD patients, although the underlying mechanism remains unknown.

##### Parkinson’s Disease

Parkinson’s disease (PD) is an adult-onset neurological movement disorder with a progressive degeneration of the dopaminergic neurons. At the cellular level, PD is characterized by the accumulation of α-synuclein in large masses called Lewy bodies [63]. Both genetic and toxicant *C. elegans* models of PD have been derived [64]. One of the genetic models of PD is generated by expressing the human *SNCA/PARK* gene, which encodes α-synuclein [65]. In this PD model, the expression of α-synuclein causes the loss of the dopaminergic neurons, deficits in dopamine-dependent behavior, and decreased dopamine levels. One study shows that blueberry extracts attenuate α-synuclein protein expression, and lower the expression of *sir-2.1/Sirt1* [66]. These observations suggest that a reduced SIR-2.1/SIRT1 activity may be beneficial to the attenuation of α-synuclein.

**Figure 5:**
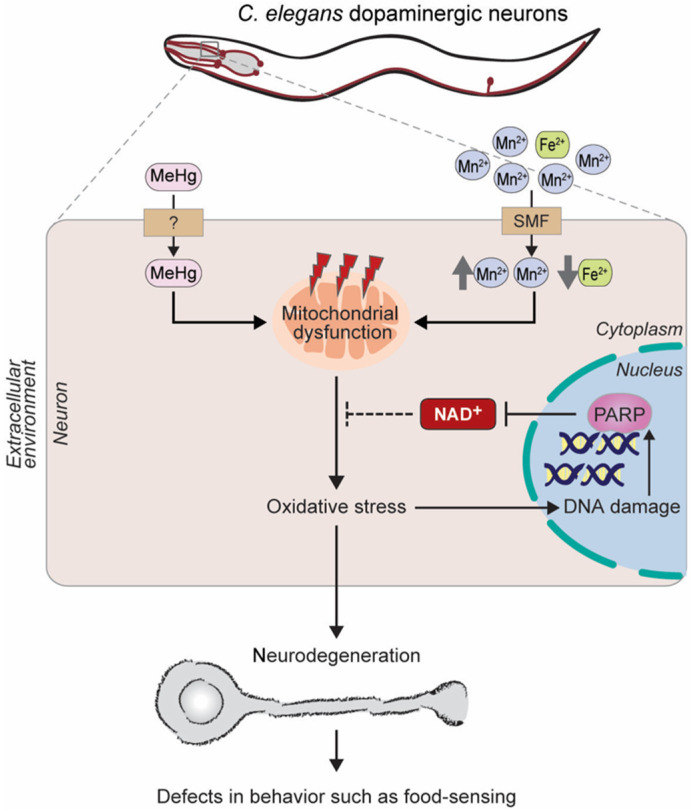
*C. elegans* models of Parkinson’s disease with chronic heavy metals treatments. Methylmercury (MeHg) and excess manganese (Mn^2+^) lead to a dopaminergic neuronal loss resembling Parkinson’s disease. (Left) Methylmercury is transported across the membrane into the dopaminergic neuron and induces mitochondrial dysfunction, oxidative stress, and, finally, neurodegeneration. (Right) Excess manganese hinders iron transport, and iron deficiency causes mitochondrial dysfunction, oxidative stress, and DNA damage. PARPs are activated to prevent DNA damage, but an excessive PARP activity leads to NAD^+^ depletion, which presumably fails to reduce oxidative stress, and eventually induces dopaminergic neurodegeneration. SMF, divalent cation transporter.

Exposure to heavy metals, such as methylmercury or excess manganese, also makes a toxicant model of PD because it is linked to a dopaminergic dysfunction in *C. elegans* and mammals [67,68,69,70,71]. Exposure to methylmercury or chronic exposure to manganese in low doses causes the depletion of the cellular NAD^+^ levels, mitochondrial dysfunction, and oxidative stress [72]. It is possible that high levels of ROS induce DNA damage and, in turn, trigger the activity of the DNA damage response protein, PARP, which likely causes the depletion of the NAD^+^ levels (Figure 5). Interestingly, NAD^+^ pretreatment decreases methylmercury-induced oxidative stress, dopaminergic toxicity, and behavioral deficits [72]. It would be interesting to investigate how the global changes of mitochondrial dysfunction and oxidative stress cause dopaminergic neuron-specific defects. A cell-specific visualization study of NAD^+^ levels would help explore the precise mechanisms.

#### 2.2.3. Chemotherapeutic Agents

Chemotherapy-induced peripheral neuropathy (CIPN) is one of the most common disorders caused by cancer treatments, affecting up to 80% of cancer patients [73]. CIPN pathology is a “dying back” axon degeneration of the distal nerve endings [74]. Taxol is used widely as a chemotherapeutic agent, even though it has been shown to induce axonal degeneration in cancer patients and in experimental models [75]. Axon degeneration in the form of axonal swelling, thinning, and gaps is seen in elderly worms and is significantly greater in taxol-treated animals [76].

*C. elegans* treated with taxol shows a significant growth retardation. This defect is reversed by the expression of the non-nuclear-localized mutant form of the mouse *Nmnat1* gene. In addition, taxol dramatically reduces the basal expression of a mitoUPR (mitochondrial Unfolded Protein Response) reporter, serving as a marker of mitochondrial proteostasis, and it was restored by the expression of non-nuclear mouse NMNAT1 [76]. Thus, NMNAT is suggested to provide protection from a taxol-induced axonal pathology. These observations suggest that NMNAT may ameliorate mitochondrial proteostasis stress and protect neurons from an axonal damage.

## 3. Regulation of Axon Regeneration in *C. elegans*

The laser-assisted axotomy technique has established *C. elegans* as a valuable model to study the molecular mechanisms of axon regeneration [77]. Transgenic animals with fluorescently labeled single neurons are subjected to laser axotomy, and then the regenerating axons are analyzed. Laser severing of the *C. elegans* axon does not cause neuronal death but does cause severe axonal damage. Although a calcium surge is often neurotoxic, a proper calcium influx following axonal damage is considered to be beneficial because it triggers injury responses, including robust axon regeneration [78,79,80,81]. This neuronal injury response contributes to the recovery of the functional impairments caused by the injury. For example, the axonal injury of motor neurons causes locomotory defects, and regenerating this injured axon is likely required to recover the animal’s locomotion [77].

### 3.1. Inhibitory Role of NMNAT and NADS in Axon Regeneration

**Figure 6:**
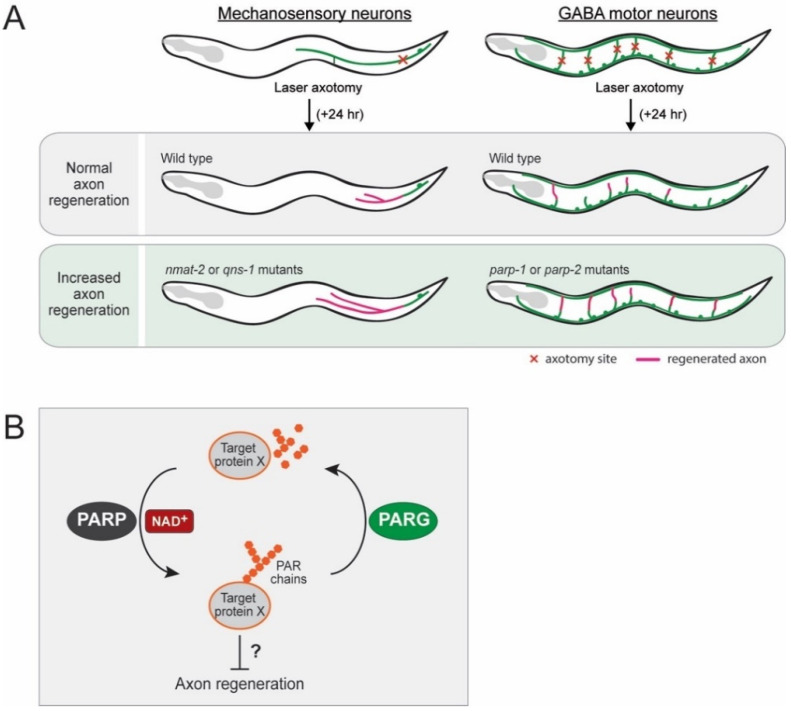
NAD^+^ regulating axon regeneration. (**A**) Axon regeneration in mechanosensory neurons is increased in *nmat-2* and *qns-1* mutants, and axon regeneration in GABA motor neurons is increased in *parp-1* and *parp-2* mutants. Thus, these genes play an inhibitory role in axon regeneration. (**B**) Speculation of the relationship between NAD^+^ levels and the PARP–PARG pathway in inhibiting axon regeneration. By utilizing NAD^+^, PARP proteins synthesize long chains of poly(ADP-ribose) (PAR) on target protein X that may inhibit axon regeneration.

This laser axotomy paradigm enables the screening of many genetic regulators controlling axon regeneration. Yishi Jin and Andrew D. Chisholm laboratories have pioneered this technique in *C. elegans* and screened numerous genes involving axon regeneration of the mechanosensory neurons [15,82]. One of their findings shows that NAD^+^ biosynthesis likely inhibits axon regeneration [15]. They have examined loss-of-function mutants of each gene encoding enzymes in the NAD^+^ salvage pathways, such as the *C. elegans* orthologs of *NMNAT* (*nmat-1* and *nmat-2*), *NADS* (*qns-1*), nicotinamide riboside kinase (*NRK*) (*nmrk-1*), nicotinate phosphoribosyltransferase (*NAPRT*) (*nprt-1*), and nicotinamidase (*pnc-1* and *pnc-2*) (Figure 2C). Among them, three independent *nmat-2* mutants and a *qns-1* mutant show increased axon regeneration following the injury of mechanosensory neurons, indicating that *nmat-2* and *qns-1*, catalyzing the terminal steps of the NAD^+^ salvage pathways, inhibit axon regeneration in *C. elegans* (Figure 6A).

Importantly, the enzymatic properties of NMAT-2 are essential for the inhibition of axon regeneration because the active site motif [83] mutation of *nmat-2* exhibits a phenotype similar to that of the null mutants of *nmat-2* [15]. Thus, the role of NMAT-2 in axon regeneration likely requires its enzymatic activity so that it may activate through a different downstream pathway. Consistent with the inhibitory role of *nmat-2*/*NMNAT* in axon regeneration in *C. elegans*, the overexpression of *NMNAT* leads to impaired sensory axon regeneration in *Drosophila* [84]. Together, these data suggest conserved roles of NMNAT in hindering axon regeneration, at least in invertebrate animal models.

Another distinct feature of NMAT-2 in axon regeneration, when compared to its neuroprotection effect, is cell autonomy. In *Drosophila* and mice, the neuroprotective effect of NMNAT is cell-autonomous [29,85]. In contrast, NMAT-2 inhibits axon regeneration via several tissues in *C. elegans*. The phenotype of the *nmat-2* null mutant is restored by an *nmat-2* transgene under the endogenous promoter. However, the transgenic expression of NMAT-2 in individual tissues of the intestine, epidermis, or neurons fails to restore the phenotype. It is restored to normal only by the combined expression of NMAT-2 in all three tissues. These results suggest that NAD^+^ may have activated inhibitory factors in neuronal and non-neuronal tissues. An intriguing possibility that has not yet been explored is that Nmnat or NAD^+^ biosynthesis plays a role in the gut–brain–skin axis regulating axon regeneration. It would be interesting to know how the interplay among NMNAT proteins expressed in different tissues affects neuronal events.

### 3.2. Inhibitory Role of PARPs in Axon Regeneration

In *C. elegans*, PARPs and poly(ADP-ribose) glycohydrolases (PARGs) show opposing effects on axon regeneration [86]. PARPs catalyze the transfer of ADP-ribose from NAD^+^ onto protein substrates (Figure 2B), whereas PARGs remove poly(ADP-ribose) from proteins [87]. The NAD^+^-consuming PARP encoding genes in *C. elegans*, *parp-1* and *parp-2*, inhibit the axon regeneration of the GABA motor neurons (Figure 6A), whereas the PARG encoding genes, *parg-1* and *parg-2*, enhance it [86,88]. Importantly, a pharmacological PARP inhibitor (A966492) significantly enhances axon regeneration when administered after injury in vivo in *C. elegans* motor neurons and in vitro in mammalian cortical neurons [86]. Treatment using a PARP inhibitor post-injury also improves the behavioral recovery in *C. elegans*, indicating that PARP inhibition after injury is sufficient to improve axon regeneration. Currently, chemical PARP inhibitors are in preclinical and clinical trials for indications, including various cancer and stroke therapies [89,90]. These results suggest that the PARG–PARP balance may determine axon regeneration by regulating the poly(ADP-ribose) levels on target proteins [91]. Thus, identifying protein substrates of PARPs and PARGs will require further investigation in the future. When considering the enhanced axon regeneration seen in both *nmat-2* and *parp-1/-2* mutant worms, it is speculated that decreased NAD^+^ levels in *nmat-2* mutants are presumably not sufficient to activate regeneration inhibitory PARP proteins (Figure 6B). Thus, it is possible that the NAD^+^ may act through the PARP pathway, which remains to be determined.

The effects of PARPs on mammalian axon regeneration have been tested in multiple studies with variable results [92,93]. Brochier and colleagues identified PARP1 as a critical mediator of multiple growth-inhibitory signals [93]. In vitro models of axonal injury reveal that the exposure to growth-inhibitory signals promotes PARP1 activation and the accumulation of PAR in neurons, which leads to an axon regeneration failure. The pharmacological inhibition or genetic depletion of PARP1 is sufficient to promote regeneration. However, Wang and colleagues found that the inhibition of PARylation fails to increase axon regeneration or improve functional recovery after an adult mammalian CNS injury [92]. These conflicts might be due to the different types of neurons being tested. The axonal regenerative effects of PARPs remain unclear.

## 4. Conclusions and Future Perspectives

**Figure 7:**
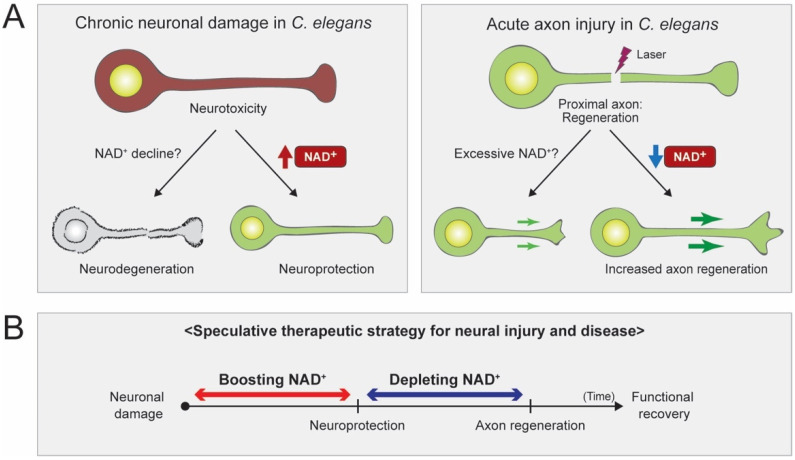
The relationship between NAD^+^ levels and the responses of damaged neurons in *C. elegans*. (**A**) High levels of NAD^+^ help protect damaged neurons, while low levels of NAD^+^ help promote axon regeneration (**B**) A proposed therapeutic strategy to improve recovery from neuronal damage by controlling NAD^+^ supplementation.

NAD^+^ biosynthesis pathways are functionally conserved in *C. elegans*, and emerging evidence is revealing the critical role that NAD^+^ plays in neuronal protection and axon regeneration. The effects of NAD^+^ have been studied in various neuronal damage models of *C. elegans.* Unlike the mammalian Wallerian degeneration paradigm, the sustained high levels of NAD^+^ show no effects in inhibiting or delaying axon degeneration in an acute injury model of *C. elegans.* In chronic injury models, however, high levels of NAD^+^ influence neuronal protection, which presumably helps reduce the oxidative stress produced by the cytosolic calcium surge and mitochondrial dysfunction (Figure 7A). The *C. elegans* laser-assisted axotomy paradigm enables the screening of many genes in the NAD^+^ pathways. All known components in NAD^+^ salvage pathways and several NAD^+^ consuming enzymes have been tested. In sensory neurons, NMNAT/NMAT-2 and NADS/QNS-1 are found to inhibit axon regeneration, suggesting that decreased levels of NAD^+^ promote axon regeneration (Figure 7A). In motor neurons, PARPs inhibit axon regeneration, whereas PARGs promote it. It is speculated that PARP proteins are activated by high levels of NAD^+^ and that they activate regeneration inhibitory target proteins by PARylation (Figure 6B). Nonetheless, given the inhibitory role of NMNAT/NMAT-2 and NADS/QNS-1 in sensory axon regeneration, it is necessary to issue a caution before manipulating NAD^+^ levels as a therapeutic strategy. As we come to understand NAD^+^ biology in greater detail, we may need to regulate the NAD^+^ levels in proper time frames and in specific tissues. For example, after neuronal damage, the treatment of high-dose NAD^+^ may help protect damaged neurons, but later, depleting NAD^+^ may help regenerate axons (Figure 7B). In the near future, a precise understanding of NAD^+^ biology may allow the development of NAD^+^-based therapeutic strategies for various neuronal damage conditions.
